# Cytotoxic Evaluation, Molecular Docking, and 2D-QSAR Studies of Dihydropyrimidinone Derivatives as Potential Anticancer Agents

**DOI:** 10.1155/2022/7715689

**Published:** 2022-04-25

**Authors:** Reem Altaf, Humaira Nadeem, Umair Ilyas, Jamshed Iqbal, Rehan Zafar Paracha, Hajra Zafar, Ana Cláudia Paiva-Santos, Muhammad Sulaiman, Faisal Raza

**Affiliations:** ^1^Department of Pharmaceutical Chemistry, Faculty of Pharmaceutical Sciences, Riphah International University, Islamabad 44000, Pakistan; ^2^Department of Pharmaceutical Sciences, Iqra University Islamabad Campus, Islamabad, 44000, Pakistan; ^3^Department of Pharmaceutics, Faculty of Pharmaceutical Sciences, Riphah International University, Islamabad, Pakistan; ^4^Centre for Advanced Drug Research, COMSATS University Islamabad, Abbottabad Campus, Abbottabad, Pakistan; ^5^Research Center for Modeling & Simulation (RCMS), National University of Sciences and Technology (NUST), Islamabad, Pakistan; ^6^School of Pharmacy, Shanghai Jiao Tong University, 800 Dongchuan, Road, Shanghai 200240, China; ^7^Department of Pharmaceutical Technology, Faculty of Pharmacy, University of Coimbra, 3000-548 Coimbra, Portugal; ^8^REQUIMTE/LAQV, Group of Pharmaceutical Technology, Faculty of Pharmacy, University of Coimbra, 3000-548 Coimbra, Portugal; ^9^Faculty of Pharmacy, Capital University of Science & Technology, Islamabad, Pakistan

## Abstract

The diverse pharmacological role of dihydropyrimidinone scaffold has made it to be an interesting drug target. Because of the high incidence and mortality rate of breast cancer, there is a dire need of discovering new pharmacotherapeutic agents in managing this disease. A series of twenty-two derivatives of *6-(chloromethyl)-4-(4-hydroxyphenyl)-2-oxo-1,2,3,4-tetrahydropyrimidine-5-carboxylate (3a-3k)* and *ethyl 6-(chloromethyl)-4-(2-hydroxyphenyl)-2-oxo-1,2,3,4-tetrahydropyrimidine-5-carboxylate (4a-4k)* synthesized in a previous study were evaluated for their anticancer potential against breast cancer cell line. Molecular docking studies were performed to analyze the binding mode and interaction pattern of these compounds against nine breast cancer target proteins. The *in vitro* cell proliferation assay was performed against the breast cancer cell line MCF-7. The structure activity relationship of these compounds was further studied using QSARINS. Among nine proteins, the docking analysis revealed efficient binding of compounds 4f, 4e, 3e, 4g, and 4h against all target proteins. The *in vitro* cytotoxic assay revealed significant anticancer activity of compound 4f having IC_50_ of 2.15 *μ*M. The compounds 4e, 3e, 4g, and 4h also showed anticancer activities with IC_50_ of 2.401, 2.41, 2.47 and 2.33 *μ*M, respectively. The standard tamoxifen showed IC_50_ 1.88 *μ*M. The 2D qualitative structure-activity relationship (QSAR) analysis was also carried out to identify potential breast cancer targets through QSARINS. The final QSAR equation revealed good predictivity and statistical validation *R*^2^ and *Q*^2^ values for the model obtained from QSARINS was 0.98 and 0.97, respectively. The active compounds showed very good anticancer activities, and the binding analysis has revealed stable hydrogen bonding of these compounds with the target proteins. Moreover, the QSAR analysis has predicted useful information on the structural requirement of these compounds as anticancer agents with the importance of topological and autocorrelated descriptors in effecting the cancer activities.

## 1. Introduction

Many of the pharmacologically active natural and synthetic compounds are composed of the heterocyclic nucleus. The derivatives of these agents containing nitrogen, oxygen, and sulphur atoms act as an important scaffold in drug designing. They are also an integral part of nucleic acid base pairs DNA and RNA such as purines and pyrimidines [1].

Most of the alkaloids isolated from marine sources showed significant pharmacological properties which consisted of dihydropyrimidine nucleus. Batzelladine alkaloids A and B are one of these alkaloids isolated from marine sources and act as potent inhibitors of HIV gp-120-CD4. This extended their application in pharmaceutical industry after the identification of another novel cell permeable molecule, 4-(3-hydroxyphenyl)-2-thione derivative, also called manostrol, as anticancer agent. The anticancer activity of manostrol depends on a new mechanism of affecting cell division by specific and reversible inhibition of mitotic kinesis motility without targeting tubulin [2]. The inhibitory action has shown to be on human kinesin Eg5 which causes mitotic arrest followed by apoptosis. This motor protein causes mitotic spindle formation. Other possible targets of these moieties have also been studied including centrin, calcium channels, and topoisomerase I [3]. Analogs of manostrol such as oxomonastrol, thio, and 3,4-methylenedoxy derivatives were developed, and their activity against HT-29 cancer cell lines were tested. Various other synthetic analogs L-771,688 and SQ 32926 have also been developed [1].

Since pyrimidine derivatives shows significant pharmacological activities and are essential constituents of living nature. Biginelli compounds have gained interest since last two decades because of their structural similarity with the clinically active dihydropyrimidine. These compounds are called as the esters of 6*-methyl-2-oxo-4- phenyl-1,2,3,4-tetrahydropyrimidine-5-carboxylic acid* and were first synthesized by Pietro Biginelli by the condensation reaction of *β*-ketoesters, aryl aldehydes, and urea under acidic condition through one pot three component synthesis [4].

Manostrol is one of the most studied Biginelli adducts because of its promising anticancer activities providing an inspiration for the design of new compounds. Several manostrol analogs have shown potent anticancer activities against MCF-7 breast cancer cell lines. Globally, breast cancer has been diagnosed as the most commonly diagnosed malignancy having the highest incidence rate of mortality in women [5]. The progression of breast cancer is associated with several factors such as age, personal history of breast cancer, reproductive, environmental, and genetic factors. Prognostic factors can be used to predict the course and clinical outcome of breast cancer. These include ER, PR, Ki-67, and HER-2. Other factors that can be used to predict prognosis include cyclin E, cyclin D1, and cathepsin D but are not measured routinely. The status of progesterone receptor, estrogen receptor, and the human epidermal growth factor receptor 2 basically determines the scheme for the treatment of breast cancer along with the clinicopathological factors such as tumor grade, size, and status of lymph node [6]. The synthesis of derivatives of different scaffold having pharmacological importance has helped us in determining the biological activities of compounds that can further be screened for disease management [7–11].

*In silico* drug designing is a form of computer-based modeling and is a rapidly developing field. The development of in silico target identifications of drugs with the strategy of fast speed and low cost is receiving a huge attention worldwide because of the limitation of throughput, accuracy and cost, experimental techniques that cannot be applied widely [12]. Major roles of in silico approaches in drug discovery processes include virtual screening, in silico ADME/T prediction and advanced methods for determining protein-ligand binding and quantitative structure-based drug design.

The *in silico* quantitative structure activity relationship (QSAR) is another approach used to find out a statistical correlation between the structure and function with the help of chemometric technique. The structure represents the substituents, properties, or descriptors of the molecules and their interaction energy fields, while the function refers to a biological and experimental outcome [13]. The chemometric procedures in QSAR refer to MLR, PLS, PCR, PCA, GA, etc. Several tools are available for the prediction of QSAR models that perform specific QSAR steps such as modelling, validation of statistics, and the descriptor generation [14]. The Open3DQSAR or PyCoMFA generates the CoMFA-like models while CORAL, a freeware software, uses a specific set of descriptors (SMILE based) to generate the QSAR PLS model [15, 16]. Another standalone freeware QSAR tool is the QSAIRNS that can help in building the QSAR MLR having the ability of model validation, data partitioning, predicting a new activity of compound, and determination of applicability [17]. Ezqsar and camb are another R-package-based tools that are available openly. They are basically used for beginners that utilize a single function to do the entire job [18].

**Figure 1:**
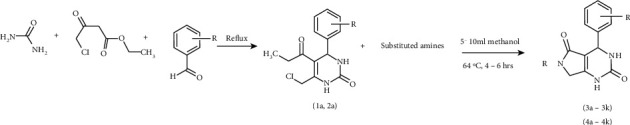
The general scheme used for the synthesis of dihydropyrimidinone derivatives.

**Figure 2:**
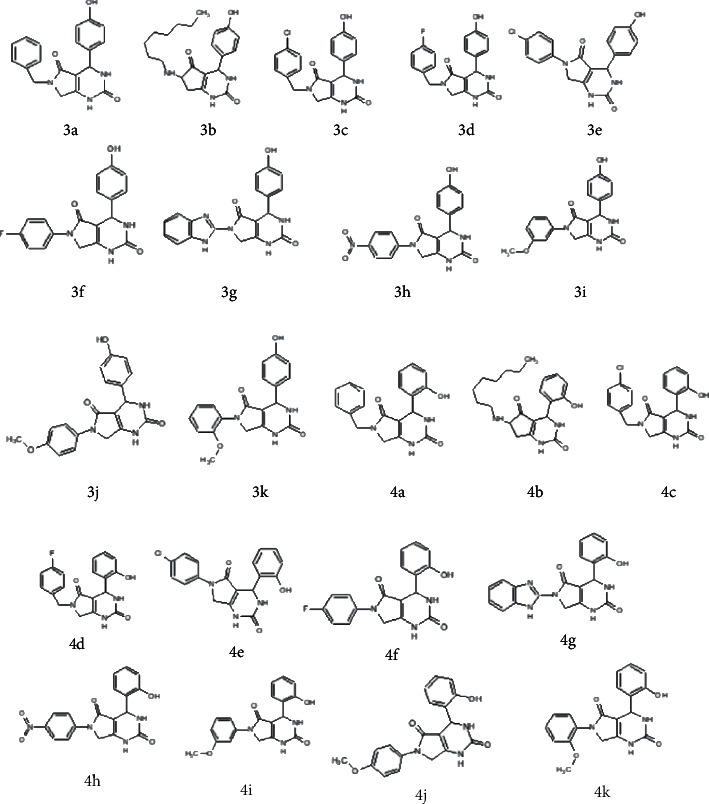
Structures of synthesized dihydropyrimidinones derivatives (3a-3k and 4a-4k).

In view of finding new potential leads with effective chemotherapeutic activities, about twenty two derivatives of *6-(chloromethyl)-4-(4-hydroxyphenyl)-2-oxo-1,2,3,4-tetrahydropyrimidine-5-carboxylate (3a-3k)* and *ethyl 6-(chloromethyl)-4-(2-hydroxyphenyl)-2-oxo-1,2,3,4-tetrahydropyrimidine-5-carboxylate (4a-4k)* were synthesized in a previous study [19] (Figure 1). A neat reaction of urea, 4-choloroethylacetoacetate, and substituted benzaldehyde were refluxed for 1 h to obtain 6-chloromethyl-DHPMs. The resulting compounds were further reacted with a series of benzyl amine derivatives in methanol. The crystals were recrystallized using ethanol. The compounds were then characterized using FT-IR, ^1^H NMR, and ^13^C NMR. The structures of the synthesized compounds are shown in Figure 2. The spectral analysis of these compounds is mentioned in supplementary Table 1. The compounds were screened for their anticancer activities. The anticancer activities of these synthesized compounds were evaluated against the breast cancer target proteins identified through system biology approach [20]. The system biology approach has helped in identifying several gene targets in better management of diseases [21]. The in silico molecular docking studies of these synthesized compounds were performed to screen for the best targets for these compounds. Furthermore, the *in vitro* efficacy of these compounds against breast cancer cell line MCF-7 was also performed to understand their antitumor effects. The in silico 2D-QSAR analysis was done to evaluate the structure activity relationship of synthesized compounds by QSARINS [22]. This was done to analyze the predicitivity and stability of models and the role of essential descriptors generated from both models.

## 2. Methodology

### 2.1. *In Silico* Chemoinformatics Analysis of Synthesized Ligands

The synthesized chemical structures were drawn in ChemBioDraw Ultra 14.0 and energy minimized using ChemBio3D Ultra 14.0. The Molsoft tool (http://www.molsoft.com/) was used to evaluate the basic chemical properties such as molecular weight (g/mol), hydrogen bond acceptor (HBA), hydrogen bond acceptor (HBA), Log*P*, molecular volume (A3), polarizibility, and drug likeness. Moreover, the ADMET properties were also evaluated using online pkCSM tool. The tool is used to predict the pharmacokinetics, drug likeness, and medicinal chemistry aspect of small molecules. The compounds having molecular weight < 500 g/mol, hydrogen bond donor < 5, hydrogen bond acceptor < 10, and number of rotatable bonds < 10 are drug likable compounds. The server also helps in identifying the absorption parameters such as the water solubility and intestinal absorption as well as skin permeation. The distribution properties such as blood brain barrier permeation and CNS permeation were also calculated. The total renal clearance and the toxicity profiling including Ames test, hepatotoxicity, and skin sensitivity was also evaluated. The ligand and lipophilic ligand efficiency (LE and LLE) as well as lipophilicity-corrected ligand efficiency (LELP) values were predicted using Data Warrior tool [23].

### 2.2. Molecular Docking Studies

PyRx docking software, an open source software, was used to identify the best target proteins for the proposed compounds [24]. Several libraries of compounds can be screened for potential target identification using PyRx, starting from job preparation to submission and analysis of results. PyRx is an easy to use and a valuable tool for Computer-Aided Drug Design and has a docking wizard AutoDock Vina. The visual analysis of results in PyRx is based on embedded Python Molecular Viewer (ePMV), and the results are stored in a built-in SQLite database.

### 2.3. Selection of Breast Target Proteins

The target proteins identified through system biology approach were used in order to study the protein-ligand interaction of these proteins with the synthesized compounds [20]. The differentially expressed breast cancer genes were identified through extensive data mapping, and functional enrichment analysis was performed to screen the differentially expressed genes between breast tumor cells and treated tissues. Moreover, the interactions of these genes with several other proteins involved in breast cancer progression were studied. The shortlisted genes showed essential role in the progression of breast cancer. All the source proteins and the target proteins were shortlisted in order to identify the best target for these compounds. These proteins include ESR, PR, BRCA1, BRCA2, AKR1C2, HER2, CTNNB1, PLAUR, and RHEB.

### 2.4. Preparation of Proteins

Protein Data Bank was used to retrieve the atomic coordinates of proteins ESR (PDB ID =1L2I), PR (PDB ID = 1A28), BRCA1 (PDB ID = 4IGK), BRCA2 (PDB ID = 3EU7), AKR1C2 (PDB ID = 4JTR), HER2 (PDB ID = 1N8Z), CTNNB1 (PDB ID = 3SL9), PLAUR (PDB ID = 2FD6), and RHEB (PDB ID = 3T5G). The details of all proteins are mentioned in Table 1. All the proteins obtained from Protein Data Bank contained water molecules and the original ligands. For the preparation of protein structures, cocrystallized ligand and any water molecules that were present were removed using MGL Tools-1.5.6, nonpolar hydrogen bonds merged, AD4.2 type and Gasteiger charges were assigned, and proteins were saved in .pdbqt format.

### 2.5. Active Site Prediction

DOGSITESCORER was used to identify the active sites of the proteins from the 3D coordinates of the receptor. DOGSITESCORER is an automated tool for pocket prediction based on 3D structure of protein and calculates the druggability of protein cavities [34]. For the prediction of druggability of pockets, the supervised machine learning technique (SVM) is utilized that predicts the potential pocket and describes them through descriptors. The site provides a druggability score between 0 and 1 showing the higher the score, the more the pocket is druggable. PyMOL was used to visualize the active site of target proteins and the residues involved [35].

### 2.6. Preparation of Ligand

The structure of ligands was drawn using ChemBioDraw Ultra 14.0, and energy was minimized using MM2 with the help of ChemBio3D Ultra 14.0. The structures were saved in PDB format for AutoDock compatibility. The ligand.pdb files were converted to ligand.pdbqt format using MGL Tools-1.5.6 (The Scripps Research Institute).

### 2.7. AutoDock Run

The protein ligand binding was analyzed with the help of PyRx tool linked with AutoDock Vina in order to find the correct conformation and configuration of the ligands having the minimum energy structure. The grid centers were positioned on the active binding sites of both proteins, and the docked complexes were examined on the basis of their binding affinities (kcal/mol) and interaction patterns.

### 2.8. Analysis of Binding Affinity

The boxplot function in R-4.0.2 package was used to perform the scoring analysis of each protein with the synthesized compounds [36]. For interaction analysis, the Discovery Studio Visualizer Software, Version 4.0 (http://www.accelrys.com) was used to study the binding modes of synthesized compounds with the target proteins.

### 2.9. *In Vitro* Breast Cancer Activities of Synthesized Compounds

The anticancer activity of the synthesized compounds was determined against human breast (MCF-7) cancer cell line. The MCF-7 (ATCC® HTB-22™) cell lines were gifted by Dr Syed Shahzad ul Hussan from Lahore University of Management Sciences (LUMS). The cells were cryopreserved at -196°C. The cells were grown in RPMI (Roswell Park Memorial Institute Medium) supplemented with 10% fetal bovine serum (FBS) and 1% penicillin/streptomycin purchased from Gibco, USA. The cultures were maintained in 5% CO_2_ atmosphere and a humidified incubator at 37°C. The different concentrations of synthesized compounds were used to assess the anticancer activity. 3-(4,5-Dimethylthiazol-2-yl)-2,5-diphenyl tetrazolium bromide (MTT) (Sigma) assay was used as described by Mosmann with a slight modification of 72 h of incubation [37]. A spectrophotometer at 520 nm was used to read the assay plates. A dose-response curve was plotted from the data generated to evaluate the concentration of tested compounds required to kill 50% of cell population (IC_50_). The compounds having % inhibition less than 50% are considered inactive.

### 2.10. QSAR Studies

The QSARINS software was used to generate models according to OECD standards (Worth et al. 2007).

### 2.11. Molecular Descriptor Generations

The PaDEL descriptor software was used to generate the quantum molecular descriptors and to calculate the additional energy, where a total of 1875 descriptors were calculated. The use of all the available descriptors would be, however, difficult to calculate the models; hence, few descriptors per model were used to reduce the computation time and to explore all the combinations with the help of all subset technique. The model generation was run for up to 8 variables to see the effect of addition of new descriptor on the quality of model.

### 2.12. Data Division

The datasets were divided in a 4 : 1 ratio having both training sets and test sets. The training set constituted of 70% while the test set is 30% of the data according to the Kennard-Stone algorithm method.

### 2.13. Model Building and Validation

The genetic algorithm (GA) technique was employed in which the most appropriate descriptors were selected to develop models based on large number of descriptors. The MLR model was obtained by the ordinary least squares (OLS) algorithm [22]. Twenty models were generated using up to 8 different descriptors, and the best model was shortlisted according to the lowest lack of fit (LOF) value.

### 2.14. Internal Validation

The validation of model was done by OECD principle which states that the model should have a definite endpoint, a clear applicability domain, an ambiguous algorithm, appropriate measure of robustness and predictivity, and a systematic explanation [38].

### 2.15. Cross Validation

For cross validation (CV), the Q^2^_LOO_ criteria were employed by iteratively removing from the dataset one compound while calculating the model with the rest of the compounds. The following parameters were considered to assess the quality of model:

*R* ^2^: highest value corresponds to the quality of the model, Q^2^_LOO_: highest values should be equal to *R*^2^, *R*^2^-Q^2^_LOO_: lower value indicates the stability of model, RMSE: value is low and close to training dataset, and other prediction methods.

Another method was used for cross validation, i.e., Leaving Many Out (LMO) allowing the study of compounds by excluding a large number of compounds. The stability of model was based on calculated values of *R*^2^ and *Q*^2^ (LMO), and their averages are close to *R*^2^ and *Q*^2^_LOO_ values of the model.

### 2.16. y-Scrambling

The y-scrambling procedure was applied to validate that the generated model was not as a result of chance correlation. The responses were scuffled as to there be no correlation with the descriptors causing the performance of the models to decay drastically. For a good quality model, the *R*^2^ and *Q*^2^ values and their averages should be less than the values of the model.

### 2.17. External Validation

The generated model was then assessed for its performance by different measures such as RMSE external, *Q*^2^-F1, *Q*^2^-F2, *Q*^2^-F3, *r*^2^M, Δ*r*^2^_*m*_, and CCC.

### 2.18. Applicability Domain

The domain of applicability was evaluated to confirm the consistency of the model within the chemical space it was developed [39]. The leverage approach was used, and the William's plot was generated between the standardized residuals and leverages.

## 3. Results

### 3.1. Pharmacokinetic Analysis

#### 3.1.1. Drug-Likeness Properties

The drug-likeness properties were validated by evaluating the chemical properties of synthesized compounds and analyzing the Lipinski Rule. For drug absorption, the polar surface area (PSA) parameter is a significant tool and the molecular lipophilicity and molar refractivity values relate to protein binding and bioavailability. For compounds to be drug like the molar refractivity should be 40-130 cm, PSA < 89 Å, and molecular weight 160-480 g/mol.  Table 2 shows the drug-likeness properties of synthesized compounds justifying a strong correlation with the standard values.

### 3.2. ADMET Studies of Synthesized Compounds

The pharmacokinetic ADMET properties were evaluated to assess the effectiveness of the synthesized compounds. The compounds having good pharmacokinetic properties and better activities are considered in the drug discovery and development. To evaluate the pharmacokinetic properties, the pkCSM tool was used. The water and intestinal solubility (log mol/L, % absorbed) and the skin permeability (LogKp) predicted values revealed efficient absorption of these compounds as well as efficient skin permeability as compared to standard value (>30% abs and -2.5 LogKp). Effective absorption of compound leads to effective potency due to passive penetration to reach the target molecule. All the compounds showed poor permeability to the blood brain barrier when compared to standard value (>0.3 to <-1), and the compounds having <-1 are considered poorly distributed in the brain. However, all the compounds showed good penetration to the CNS having LogPS > −2 when compared to standard value (>-2 to <-3 LogPS). The compounds having LogPS < −3 are impossible to cross in the CNS. The toxicity profiling revealed all the compounds are nonmutagenic and nontoxic except for compounds 3a, 3g, 3h, and 4h. All the compounds also showed hepatotoxicity but no skin sensitization was revealed (Table 3).

### 3.3. Lead Optimization

Further drug-likeness properties of all compounds such as ligand efficiency (LE), lipophilic ligand efficiency (LLE), and lipophilic-corrected ligand efficiency (LELP) values were predicted. The lipophilicity is considered to be a basic parameter to enhance structure efficiency making it from lead to drug candidate. ThecLog*P*, LE, LLE, and LELP of all compounds showed comparable results with that of standard values forLE > 0.30 kcal/mol/HA,LLE > 0.5 kcal/mol,LELP − 10<to <10, andcLog*P* < 3. All the synthetic compounds showed to have none mutagenic and irritant behavior (Table 4).

### 3.4. Molecular Docking

**Figure 3:**
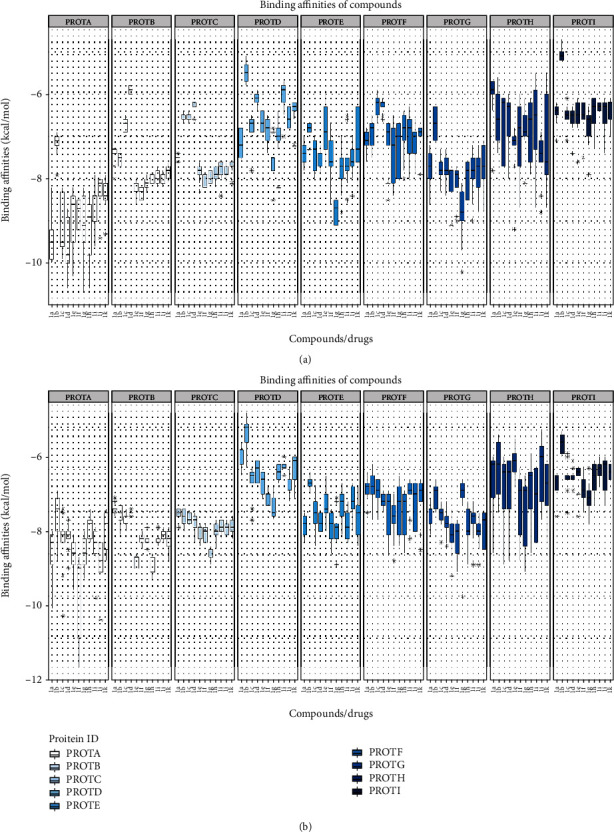
Boxplot for docking scores generated by RStudio 4.0. (a) *6-(Chloromethyl)-4-(4-hydroxyphenyl)-2-oxo-1,2,3,4-tetrahydropyrimidine-5-carboxylate derivatives (3a-3k).* (b) *Ethyl 6-(chloromethyl)-4-(2-hydroxyphenyl)-2-oxo-1,2,3,4-tetrahydropyrimidine-5-carboxylate derivatives (4a-4k).* The *y*-axis represents the docking scores while the *x*-axis shows the synthesized compounds. ProtA: CTNNB1; ProtB: BRCA1; ProtC: BRCA2; ProtD: AKR1C2; ProtE: ESR; ProtF: HER2; ProtG: PLAUR; ProtH: PR; ProtI: RHEB.

The molecular docking studies of synthesized compounds against nine target proteins were performed to analyze the best target for these compounds based on docking scores. The boxplot was generated to present the docking scores of all target proteins.  Figure 3 shows the boxplot of all synthesized compounds on the basis of their interactions with all target proteins.

In case of protein A (CTNNB1) according to the median value, the compound 4f is having the lowest median score of -11.7 with 80% of data in lower quartile and 20% in upper quartile. The compounds 4h and 4e showed the lowest median score of -10.4 and -10.3, respectively, with equal distribution of data. The compound 4k showed the median score of -10.3 with 75% of data in lower quartile and 25% in upper quartile. The compounds 3e and 3f showed the median score of -10.1 and -9.8 with 60% of data in lower quartile and 40% in upper quartile. The compounds 4g, 4i, 3g, 4j, and 4h showed median score in the range of -9.8 to -9 kcal/mol. In protein B (BRCA1), the compound 4h showed the median score of -9.3 with 90% of data in upper quartile, 4e showed -8.5 median score with equal distribution, and 4f showed median score of -9.2 with 90% of data in upper quartile; 4k and 3e showed -8.8 with equal distribution and -8.6 with 90% in upper quartile, 3f had median score of -8.5 with 80% in lower quartile and 20% in upper quartile, 4g showed -9.8, and 4i had a score of -8.4 with 90% in upper quartile. The protein C (BRCA2) also showed a similar pattern but with the median score in the range of -8.9 to -6.6. The compound 4f showed the lowest median score of -8.3 with 80% of data in lower quartile, 4e showed -8.7, and 4h showed -8.9. Similarly, the compounds 3f and 3g had the lowest median score of -8.4 and 3e -8.3 with equal distribution.

The protein D (AKR1C2) showed the median score in the range of -8.5 to -5.8 with low range and varying distribution. The binding affinities for AKR1C2 were less when compared to proteins A, B, and C. The protein E (IGFR1) had the median score ranging between -9.2 and 7 with binding affinities better than protein D and high range. In protein G (RHEB), all the compounds had median score in the range of -7.8 to -6 kcal/mol with varying distribution and high range. Similarly, the protein F (HER2) showed the lowest median score in the range of -8.8 to -6.6 with varying distribution. The protein H (PLAUR) showed the median score of -9.2 to -6.8 kcal/mol. A highest median range was observed in protein H with varying distribution. Moreover, the protein I (PR) showed the median score of -7.8 to -5.1 kcal/mol with low median range and varying distribution of data. In all the proteins, the compounds 4f and 4h showed the lowest binding scores. The docking of ligands into the active binding site of CTNNB1 showed the lowest binding scores.

### 3.5. Interaction Analysis with Target Proteins

**Figure 4:**
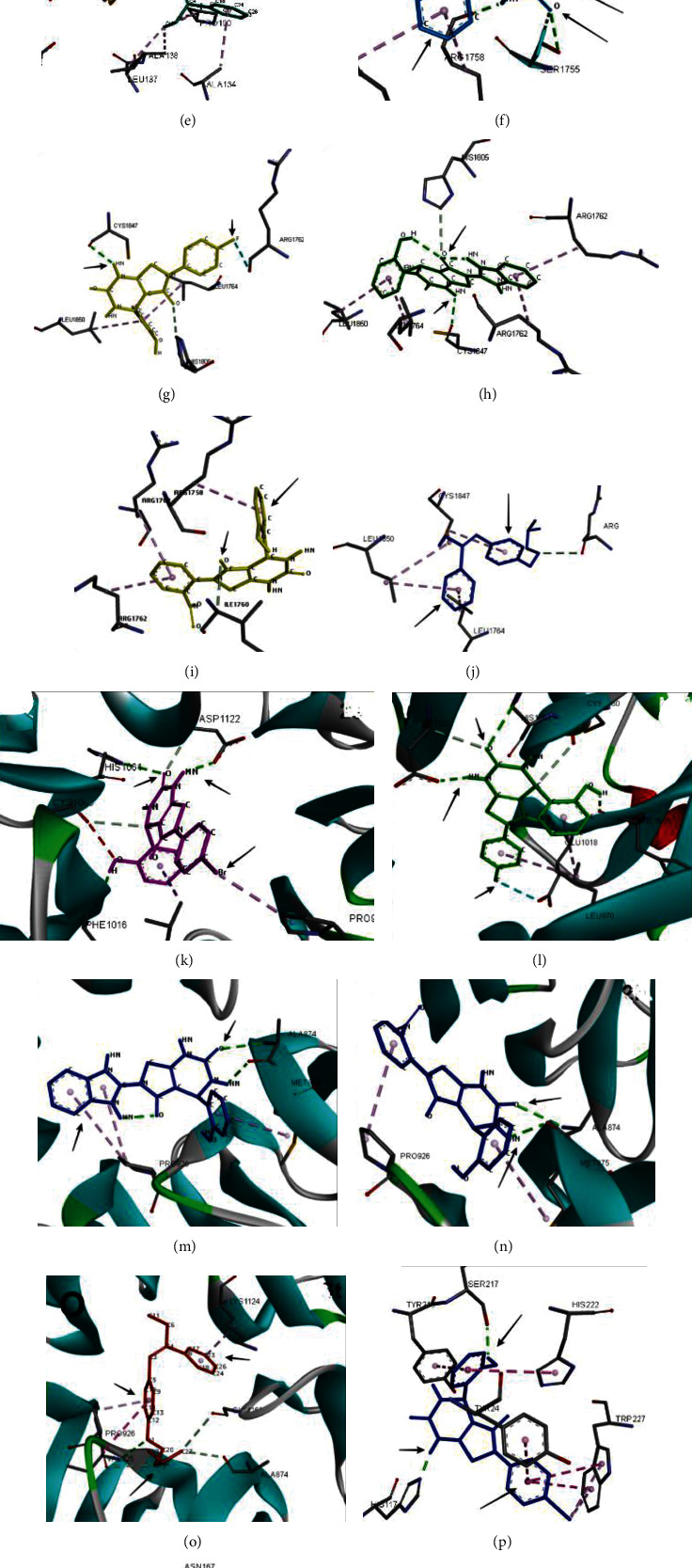
Protein-ligand interactions of target proteins with active compounds 4e, 4f, 4g, and 4h. (a–e) CTNNB1, (f–j) BRCA1, (k–o) BRCA2, and (p–t) AKR1C2.

**Figure 5:**
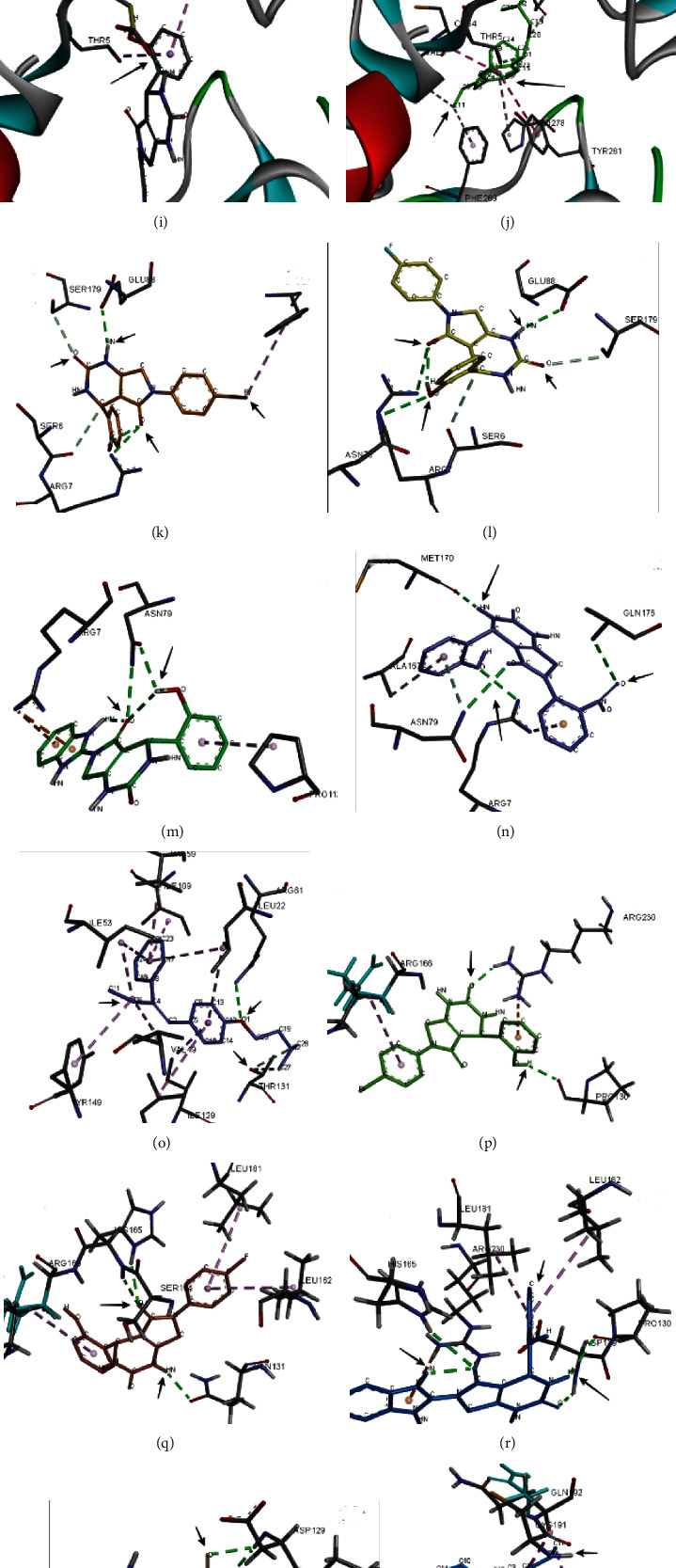
Protein-ligand interactions of target proteins with active compounds 4e, 4f, 4g, and 4h. (a–e) ESR, (f–j) HER-2, (k–o) RHEB, (p–t) PLAUR, and (u–y) PR.

The protein ligand interaction analysis was performed to study the interaction patterns of ligands with different proteins in order to find the common binding sites in proteins subjecting to new functional roles.  Figure 4 shows the binding mode of active compounds 4e, 4f, 4g, and 4h and standard against target proteins CTNNB1 (Figures 4(a)–4(e)), BRCA1 (Figures 4(f)–4(j)), BRCA2 (Figures 4(k)–4(o)), and AKR1C2 (Figures 4(p)–4(t)). Similarly, Figure 5 shows the binding mode of active compounds against target proteins ESR (Figures 5(a)–5(e)), HER-2 (Figures 5(f)–5(j)), RHEB (Figures 5(k)–5(o)), PLAUR (Figures 5(p)–5(t)), and PR (Figures 5(u)–5(y)).

Figure 4 shows the residue interactions of active compounds 4e, 4f, 4g, and 4h with the protein CTNNB1 (ProtA). These compounds showed the lowest binding scores of -10.3, -11.7, -9.8, and -10.4 kcal/mol, respectively. The interaction analysis revealed stable hydrogen bond interactions of compound 4i with ASP199, while compound 4j showed two stable hydrogen bond interactions with LEU177 and GLU176. The standard tamoxifen showed pi-alkyl with PRO100, ALA138, LEU137, LYS199, and ALA134 and amide-pi stacked interactions with VAL197 (Figures 4(a)–4(e)).

In protein BRCA1 (ProtB), stable conventional hydrogen bonding was observed in compounds 4f and 4g with CYS1847 and 4e with amino acid TYR1845, SER 1755, ARG 1758, and ILE 1760. In compound 4h, no hydrogen bonds were observed; however, pi-alkyl interactions were seen with ARG1762. The standard tamoxifen showed no hydrogen bonds, and pi-alkyl interactions were observed with LEU1764, LEU1850, and CYS1847 (Figures 4(f)–4(j)). In BRCA2 (ProtC), the compounds 4e and 4f revealed hydrogen bond interaction with ASP1122, HIS1061, and PHE1016 and 4g and 4h with ALA874. The standard tamoxifen showed hydrogen bonding with VAL925 (Figures 4(k)–4(o)). The interaction analysis of protein D (AKR1C2) showed that the compound 4e showed hydrogen bonding with SER217 and HIS117. 4f showed hydrogen bonding with TYR24 and ASN167. About four hydrogen bonding were observed in compound 4h with amino acid TYR272, ARG 276, LEU219, and SER221. The compound 4i showed hydrogen bonding with GLN190 and 2k with GLN224 (Figures 4(p)–4(t)).

In protein E (ESR), compound 4e showed 3 hydrogen bonding with SER329, TYR328, and ARG352. Similarly, compound 4f showed hydrogen bonding with GLY521 while 4g showed four hydrogen bonding with THR347, TYR537, GLY344, and GLU330, and compound 4h showed stable interactions with ASP538 and LEU539 (Figures 5(a)–5(e)). The interaction patterns of protein F (HER2) showed stable interactions of compounds 4e, 4f, 4g, and 4h with ASP8, GLY270, and THR7 along with some van der Waal interactions (Figures 5(f)–5(j)). In protein G (RHEB), the compounds 4e, 4f, and 4g showed three hydrogen bond interactions with ARG7, SER179, ASN79, and GLU88. The compound 4h showed ARG7, ASN79, and MET 170 (Figures 5(k)–5(o)). In protein H (PLAUR), the compound 4e showed hydrogen bonding with ASP697 and LYS769 and in 4f with LYS769, SER728, and GLU695. 4h showed hydrogen bonding with ILE699 and ARG766 (Figures 5(p)–5(t)). The interaction analysis of protein I (PR) is shown in Figure 5. The compound 4e showed conventional hydrogen bonding with ASP697, LYS769, and LEU755. In compound 4f, the interaction between fluorine and nitrogen group of dihydropyrimidinone was observed with amino acid SER726, GLU695, and LYS769. 4g showed hydrogen bonding with ILE699 and ARG766 (Figures 5(u)–5(y)).  Table 5 highlights the important common residues involved in interactions with the active compounds.

### 3.6. Anticancer Activity

**Figure 6:**
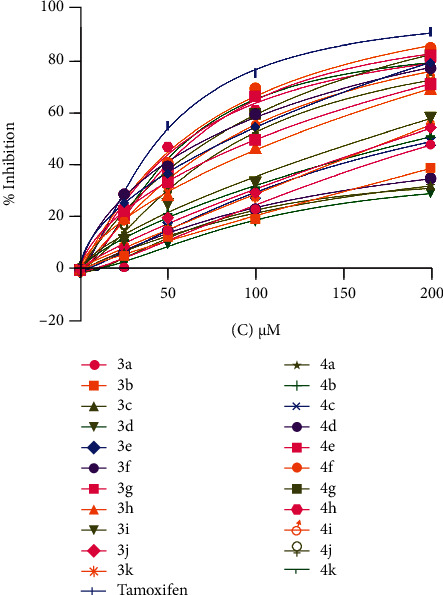
The antiproliferative effects of synthesized compounds in breast cancer cell lines (MCF-7) after 72 h treatment. The cell viability was measured by MTT assay.

In this study, the *in vitro* anticancer activity of 22 derivatives of synthesized compounds was determined against the human breast (MCF-7) cancer cell lines with the help of MTT assay (Table 6). The results revealed that the compounds having p-hydroxyl group of benzaldehyde (2) showed excellent anticancer activities when compared to standard against the breast cancer cell line. The compounds that showed more than 50% of inhibition were considered active. The compound 4f showed 85% inhibition of cells with an IC_50_ of 2.19 at 200 *μ*M concentration. The standard tamoxifen showed IC_50_ of 1.88 *μ*M. The compounds 4e and 4 g showed 82% inhibition with an IC_50_ of 2.401 and 2.47, respectively. The compound 4h also showed 80% inhibition of cells with IC_50_ of 2.33. The % inhibition of compounds 3e and 3f was 79.4 and 77.2% with IC_50_ of 2.41 *μ*M. The compounds 4k, 4i, and 4j showed up to 75% inhibition with IC_50_ of 2.40, 2.699, and 2.88, respectively. The compounds 3h, 3i, 3j, and 3k showed approximately 55% inhibition at the same concentration, while the compounds 3a, 3b, 3c, 3d, 4a, 4b, 4c, and 4d showed less than 50% of inhibition (Figure 6).

### 3.7. QSAR Studies

**Figure 7:**
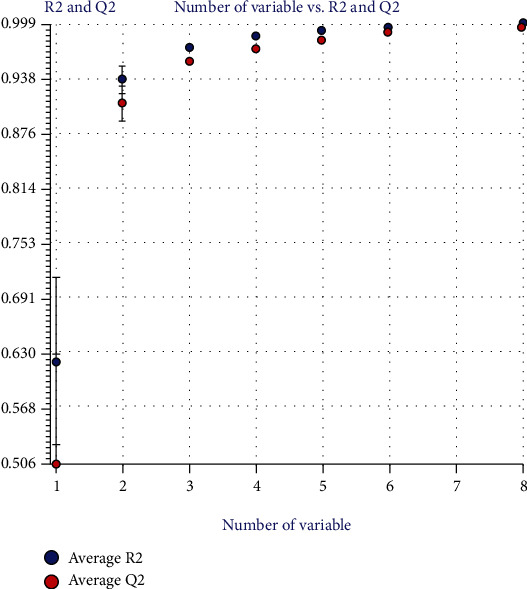
Performance of models according to different variable obtained from QSARINS.

The dataset consisting of 22 compounds was divided into training set of 15 compounds and test set of 6 compounds, where training set was used to develop the model while test set to evaluate the predictive ability of the model. Using the PaDEL software, 1872 descriptors were calculated which were then filtered using the QSARINS software. The descriptors having 80% constant values and 90% correlation were eliminated. About 1058 variables were excluded from the study based on all subset method. Several models were developed having good correlation with the response and a low multicollinearity between descriptors. The genetic algorithm–multiple linear regression (GA-MLR) method provided 4 descriptors which were then used for calculating the anticancer activities of the compounds. The average values of *R*^2^ and *Q*^2^_LOO_ (with their standard deviation) were plotted to evaluate the model performances versus the size of the developed models. It also revealed whether any overfitting in the models exists (Figure 7). The plot showed that by adding a new descriptor, the values of *R*^2^ and *Q*^2^_LOO_ increased. The model with four variables was selected based on the lowest LOF value to predict the anticancer activities.

The best MLR model equation obtained is shown below.

(1) $$\begin{array}{c} {IC}_{50}=-62.95-10.475\;MATS3i-0.144VR2\_Dzi+986.6ASP-5+3.99GGI10 \end{array}$$

**Figure 8:**
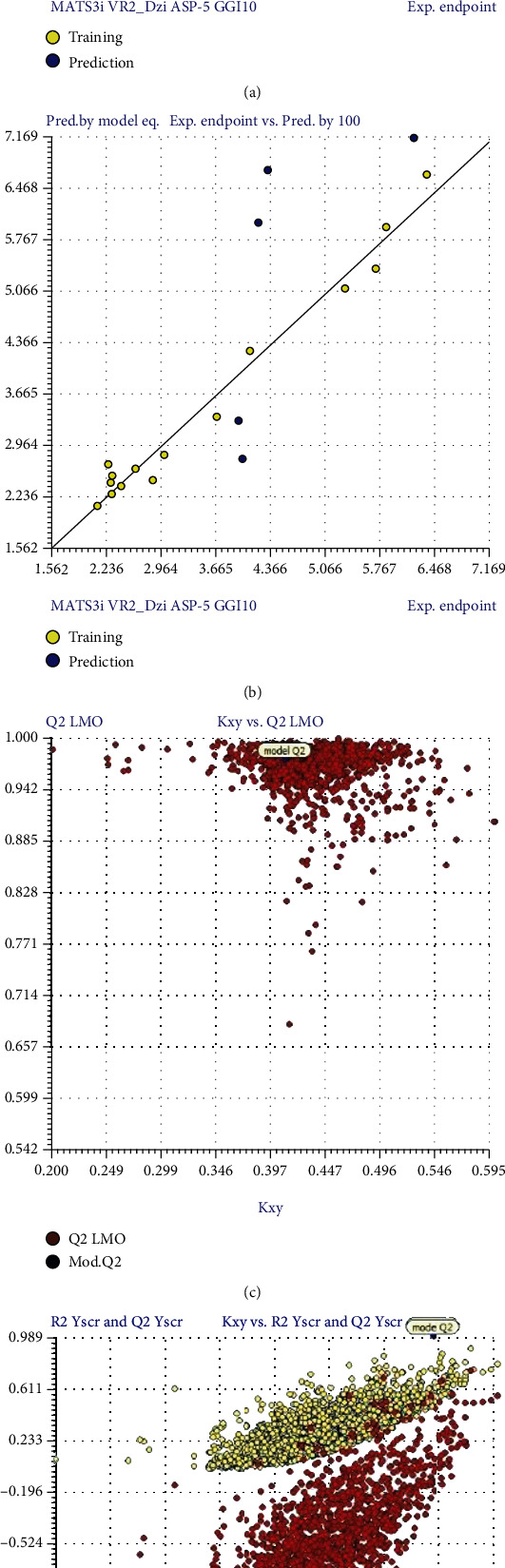
The internal validation of models through different methods. (a) The scatter plot of experimental IC_50_ versus predicted by model equation. (b) The scatter plot obtained by LOO method. (c) Plot comparing the original model with the LMO validations. (d) Plot comparing the original model with the y-scrambling model.

**Figure 9:**
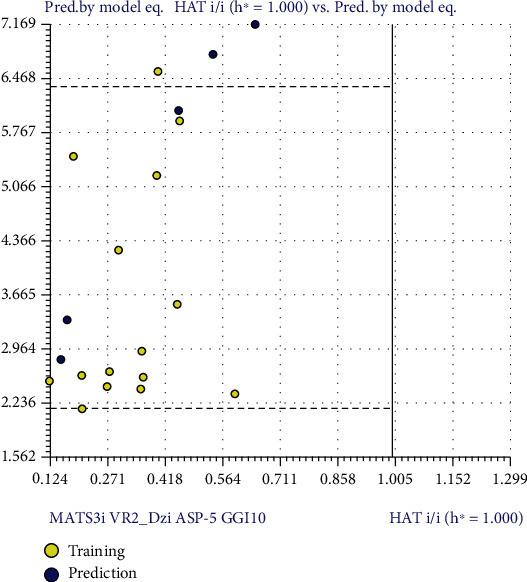
William's plot of the dataset of IC_50_ standardized against its descriptor space.

Table 7 shows the experimental IC_50_ and the results predicted by MLR model for training set.  Table 8 shows the Pearson correlation matrix which describes that a low value in coefficient (<0.7) between each pair of descriptor shows no significant multicollinearity among descriptors in the developed model. The internal validation of the model that is the scatter plot, scatter plot by LOO, scatter plot by LMO, and y-scrambling predicted the reliability of the model as shown in Figure 8. The applicability domain also defined the reliability of the model (Figure 9).

## 4. Discussion

Breast cancer pathogenesis and progression has been studied extensively with the discovery of several agents that have proved potential in the management of this disease. However, till date, the incidence rate of breast cancer is still significant and requires further strategies to combat the mortality and morbidity rate. This study uses the computational technology to identify the breast cancer targets for the synthesized compounds that can have potential role as breast cancer activities.

The *in silico* ADMET and lead optimization studies revealed all the compounds to be nonmutagenic and noncarcinogenic having drug-like properties. The results depicted compounds may act as therapeutically active against target proteins. All the synthesized compounds also followed the Lipinski Rule of 5 having HBA < 10 and HBD < 5, Log*P* < 500 g/mol. The increase number of HBA and HBD results in poor permeation. The molecular docking analysis was performed to analyze the binding of synthesized compounds with the identified target proteins. In the protein-ligand docking analysis, when comparing the binding energies and the interaction pattern, all the compounds showed the lowest binding affinity towards the target protein A (CTNNB1). The interaction analysis revealed stable hydrogen bond interactions of compound 4i with ASP199, while compound 4j showed two stable hydrogen bond interactions with LEU177 and GLU176. The standard tamoxifen showed pi-alkyl with PRO100, ALA138, LEU137, LYS199, and ALA134 and amide-pi stacked interactions with VAL197. The energy scores revealed efficient binding of these compounds with the target proteins. All the other proteins also showed efficient binding and interaction pattern, and the common amino acid residues involved in interaction are mentioned in Table 5.

The breast cancer activities of all the synthesized compounds were performed against the cell line MCF-7. The MCF-7 cell line is considered estrogen receptor- (ER-) positive and progesterone receptor- (PR-) positive expressing high level of Er*α* transcripts [40, 41]. The epidermal growth factor receptor (EGFR) and the human epidermal growth factor receptor-2 (HER2) are also present in MCF-7 cells [40]. The MCF-7 cells are also positive for *β*-catenin [42]. Due to the expression of these proteins by MCF-7 cell line, it was used to analyze the role of synthesized compounds as cytotoxic agents. It was observed that the activities of compounds 4f, 4h, and 4e were greater than all the compounds and were due to the –F, NO_2_, and –Br aniline groups with fluorine having the most potent activity due to its high electronegative nature. By replacing the groups with benzylamine (3a and 4a), –Br benzylamine (3c and 4c), and –F benzylamine (3d and 4d), the activity dropped significantly suggesting the more cytotoxic activities of aniline derivatives when compared to benzylamine derivatives. The compounds 3e, 3f, and 3g also showed better activities due to the aniline nature of compounds with –NO_2_ group of 3h showing the least activity. The benzimidazole moiety of compounds 3g and 4 g also showed effective nature of this molecule. The compounds 4k, 4i, and 4j also showed good activities having the anisidine moieties. The ortho anisidine showed more % inhibition than para and meta. This study was carried due to the existence of several evidences on the antiproliferative activities of dihydropyrimidinones by scientists. In a similar study, about 22 manostrol analogs were synthesized by Matias and coworkers and studied for their antiproliferative activities against five different cancer cell lines. Their result also showed stronger antiproliferative activities of their compounds against MCF-7 cancer cell line with compounds having chlorine moiety displaying significant effects on the proliferation of hepatic (HepaRG), colon (Caco-2), and breast (MCF-7) cancer cell lines [43]. Another series of 32 novel Biginelli dihydropyrimidinones were synthesized by Kumar and colleagues and were studied for their *in vitro* antioxidant and anticancer activities. The compounds exhibited significant anticancer activities against breast cancer cell line MCF-7 at 10 *μ*g concentration [44]. The cytotoxic activities of another synthesized library of dihydropyrimidinone benzopyran hybrids were evaluated for their cytotoxic activities against four human cancer cell lines A549 (lung carcinoma), MCF-7 (mammary gland adenocarcinoma), HCT-116 (colorectal carcinoma), and PANC-1 (pancreatic duct carcinoma) and showed consistent cytotoxic activities against these cell lines [45]. The antiproliferative activities of dihydropyrimidinones were also studied in another study depicting potent cytotoxic activities of dihydropyrimidinone analogs against melanoma (UACC.62), kidney (786-0), breast (MCF-7), ovarian (OVCAR03), and, particularly, colon (HT-29) cancer cell lines [46]. All the evidences support the significant role of dihydropyrimidone in breast cancer cell line. Moreover, the significant activities of these compounds against breast cancer cell line and optimum binding energies of these compounds against identified target proteins support the effectiveness of these compounds as anticancer agents.

The QSAR studies were performed by two different software to analyze the model quality and their reliability by both methods.

The model was generated by the QSARINS having the following fitting criteria:

*N* (number of compounds in the training set) = 15, *R*^2^ (coefficient of determination) = 0.989, *R*^2^_adj_ (adjusted *R*^2^) = 0.985, *s* (standard error of estimate) = 0.182, *F* (variance ratio) = 234.487, and RMSE_tr_ (Root Mean Square Error in fitting of training set) = 0.148.

According to the fitting criteria, the *R*^2^ value is 0.989 that is closer to 1 that shows a good quality model for anticancer inhibition. Moreover, the lower value of LOF and the *R*^2^_adj_ of 0.985 depicting the convenience to add a new descriptor to the model suggest no overfitting in the model. The model showed to be a good model having least amount of descriptors. The higher value of *F* (234.487) and the low value of *k*_*xx*_ (0.324) show minimum correlation between the descriptors. Similarly, the Delta *k* (0.084) and the small error on training sets (RMSE_tr_ = 0.148) showed appropriate correlation between the descriptors. The scatter plot obtained by the model equation versus the experimental IC_50_ for training set determines the availability of potential outliers (Figure 8(a)). The scatter plot detects the grouping of the data and the possibility of any outlier present.

### 4.1. Internal and External Validation of the Model

The internal validation of the model was done to check the fitting and stability of the models. The cross validation by *Leave-One-Out* (LOO) method showed good internal prediction as the *Q*^2^_LOO_ = 0.977 (variance explained by LOO) has a comparable value with *R*^2^ = 0.989. Moreover, the small error in prediction of RMSE_cv_ = 0.217 shows a robust and stable model. A plot was generated between the predicted values by LOO versus the experimental values of IC_50_ (Figure 8(b)). Another method was employed for internal validation that is *Leaving-Many-Out* (LMO) that leaves out 30% of the dataset to study the model behavior. The values of *Q*^2^_LMO_ = 0.9721 and the calculation in each iteration of LMO and their averages are comparable to the values of *R*^2^ and *Q*^2^_LOO_ of the model revealing the stability of the model.  Figure 8(c) displays the plot between the *Q*^2^_LMO_ and the correlation between descriptors and IC_50_ (*k*_*xy*_) showing that the model is a good fit having robustness and stability. The y-scrambling method was employed to determine whether the model is the result of chance correlation. For a good model with low chance of correlation, the values of *R*^2^ and *Q*^2^ and their averages *R*^2^_y−scr_ and *Q*^2^_y−scr_ should be lower than the values obtained previously. Here, *R*^2^_y−scr_ = 0.28 and *Q*^2^_y−scr_ = −0.66 that are far from the values obtained for *R*^2^ and *Q*^2^ indicating the model has not been obtained by random correlation.  Figure 8(d) shows the plot between the *R*^2^_y−scr_ and *Q*^2^_y−scr_ values against the *R*^2^ and *Q*^2^ of the model.

The external validation of the model was also performed to test the predictive ability of the model. The model showed *R*^2^_ext_ (external determination coefficient [47]): 0.97, *R*^2^_ext_: 0.6479, *Q*^2^-F1: 0.7320, *Q*^2^-F2: 0.8682, and *Q*^2^-F3 (variances explained in external prediction [48]): 0.702. The parameters were equivalent to the value of *R*^2^ model. The predictions of compound in external set are shown in Figure 8(a).

The reliability of the model is based on the compounds falling in the applicability domain (AD). The leverage (*h*) and standardized residuals were used as described by [49]. William's graph was generated to observe the compounds lying in the applicability domain of the model (Figure 9) by plotting the standardized residuals for each compound against the leverage values. In the applicability domain, a defined domain is set up constituting all the data points within the boundary for residuals having a leverage threshold of HAT *i*/*i* *h*∗ = 1.000 [50]. Most of the compounds fall in the applicability domain except for the compound 3f having value greater than critical leverage (*h* = 1.29) that can be considered as an outlier.

### 4.2. Interpretation of Descriptors

In model generated by QSARINS, 70% of the anticancer activity can be described using four descriptors. All the variables belong to 2-dimensional family (MATS3i, ASP-5, VR2, and GGI10). The descriptor GGI10 belongs to the GALVEZ family and is a topological charge index that has its origin in first ten eigenvalues. There are two categories for the GALVEZ class, that is, the topological charge index of order *n* (GGIn) and the mean topological charge index of order n (JGGIn). The “*n*” is the order of eigenvalue. The GGI10 is the topological charge index of order 10 and has shown positive correlations to the activity, suggesting an increase in value of GGI10 would augment the anticancer activities of synthesized compounds. The descriptor VR2_Dzi also belongs to topological distance matrix and is defined as the normalized Randic-like eigenvalue-based index from Barysz matrix weighted by ionization potential. The negative correlation suggests lower value is associated with the activity of compounds. The 2D-AUTO descriptor (MATS3i) is the topological structure of Moran autocorrelation of lag 3 weighted by ionization potential. It is the summation of different autocorrelation functions giving different vectors based on lengths of structural fragment. The weighted component in the descriptor is linked to the physicochemical property suggesting the association of topology of the structure with the selected property. The autocorrelation vector of lag *k* is indicative of the number of edges in the fragment, while the last character of the descriptor “*i*” shows the physicochemical property that is the ionization potential. The negative correlation of MATS3i in the model suggests unfavorable conditions associated with lag 3 weighted by ionization potential. All the descriptors were not correlated with each other.

## 5. Conclusion

In this study, 22 derivatives of *ethyl 6-(chloromethyl)-4-(4-hydroxyphenyl)-2-oxo-1,2,3,4-tetrahydropyrimidine-5-carboxylate* were evaluated for their potential for anticancer activities. The compounds 4e, 4f, 4g, and 4h showed good anticancer activities against the breast cancer cell line MCF-7 when compared to standard tamoxifen. The in silico data also revealed best binding affinity and interaction pattern of these compounds against target proteins; moreover, the lead optimization revealed that the compounds have drug-like properties and may act as a lead. The QSAR analysis was carried out to investigate the role of molecular descriptors in attributing anticancer activities of synthesized compounds. The models developed to predict the structural features of these compounds as anticancer revealed useful information about the structural requirement of these compounds suggesting the importance of topological and autocorrelated descriptors. Further, *in vitro* assays will be carried out to confirm the role of these compounds in targeting these proteins.

## Figures and Tables

**Table 1:** List of target proteins used for docking purpose.

Protein	PDB ID	Resolution (Å)	Structure title	Specie	Ref
ESR	1L2I	1.95	Human estrogen receptor alpha ligand-binding domain in complex with (R,R)-5,11-cis-diethyl-5,6,11,12-tetrahydrochrysene-2,8-diol and a glucocorticoid receptor-interacting protein 1 NR box II peptide	*Homo sapiens*	[25]
PR	1A28	1.80	Hormone-bound human progesterone receptor ligand-binding domain	*Homo sapiens*	[26]
BRCA1	4IGK	1.75	Structure of human BRCA1 BRCT in complex with ATRIP peptide	*Homo sapiens*	[27]
BRCA2	3EU7	2.20	Crystal structure of a PALB2/BRCA2 complex	*Homo sapiens*	[28]
AKR1C2	4JTR	1.30	AKR1C2 complex with ibuprofen	*Homo sapiens*	[29]
HER2	1N8Z	2.52	Crystal structure of extracellular domain of human HER2 complexed with Herceptin Fab	*Homo sapiens*	[30]
CTNNB1	3SL9	2.20	X-ray structure of beta catenin in complex with Bcl9	*Homo sapiens*	[31]
PLAUR	2FD6	1.90	Structure of human urokinase plasminogen activator in complex with urokinase receptor and an anti-upar antibody at 1.9 A	*Homo sapiens*	[32]
RHEB	3T5G	1.70	Structure of fully modified farnesylated Rheb in complex with PDE6D	*Homo sapiens*	[33]

**Table 2:** Cheminformatic properties of compounds (3a-4k).

Properties	Mol. weight (g/mol)	No. HBA	No. HBD	Mol. Log*P*	Mol. PSA (A2)	Stereo centers	Mol. Vol (A3)	Molar refractivity (cm^3^)	Surface tension (dyne/cm)	Density (g/cm^3^)	Polarizability (cm^3^)	Lipinski Rule
3a	335.13	3	3	1.41	70.35	1	234.3	93.56	77.4	1.43	37.09	Yes
3b	357.21	3	3	3.365	70.60	1	399.41	99.87	57.4	1.24	39.59	Yes
3c	369.09	6	3	2.00	81.67	1	363.31	98.39	79.5	1.51	39.0	Yes
3d	353.12	3	3	1.47	70.35	1	352.04	93.67	74.8	1.48	37.13	Yes
3e	415.05	3	4	3.01	78.73	1	363.19	95.26	84.2	1.74	37.76	Yes
3f	355.13	3	4	2.24	78.73	1	347.25	87.67	75.2	1.53	34.74	Yes
3g	363.13	3	4	0.75	89.55	3	397.93	96.82	101.3	1.62	38.38	Yes
3h	366.10	5	3	1.58	103.2	1	361.92	93.57	89.6	1.60	37.06	Yes
3i	351.12	9	3	1.49	77.24	1	364.27	93.90	75.3	1.47	37.22	Yes
3j	351.12	4	3	1.60	77.45	1	366.49	93.90	75.3	1.47	37.22	Yes
3k	351.12	4	3	1.74	77.45	1	366.56	93.90	75.3	1.47	37.22	Yes
4a	335.13	3	3	1.86	69.28	1	348.41	93.56	77.4	1.43	37.09	Yes
4b	357.21	3	3	3.97	69.53	1	401.7	99.87	57.4	1.24	39.59	Yes
4c	369.09	3	3	2.45	69.28	1	365.60	98.39	79.5	1.51	39.0	Yes
4d	353.12	3	3	1.92	69.28	1	354.32	93.67	74.8	1.48	37.13	Yes
4e	399.02	3	3	2.93	68.84	1	358.78	95.26	84.2	1.74	37.76	Yes
4f	339.10	3	3	2.16	68.84	1	342.84	87.67	75.2	1.53	34.74	Yes
4g	363.13	5	4	1.20	88.48	3	400.23	96.82	101.3	1.62	38.38	Yes
4h	366.10	5	3	2.02	102.2	1	361.92	93.57	89.6	1.60	37.06	Yes
4i	351.12	4	3	1.93	76.17	1	366.55	93.90	75.3	1.47	37.22	Yes
4j	351.12	4	3	2.18	76.38	1	368.85	93.90	75.3	1.47	37.22	Yes
4k	351.12	4	3	2.05	76.38	1	368.77	93.90	75.3	1.47	37.22	Yes

**Table 3:** ADMET properties of synthesized compounds.

	Absorption			Distribution			Excretion	Toxicity			
	WS	IS	SP	BBBP	CNSP	CYP3A4	TC	AMES toxicity	Max tolerated dose	HT	SS
3a	-3.57	94.724	-2.738	-0.854	-2.369	No	0.181	Yes	-0.238	Yes	No
3b	-3.395	93.057	-2.739	-1.09	-2.689	No	1.147	No	-0.781	Yes	No
3c	-3.639	93.342	-2.739	-1.038	-2.258	No	0.055	No	-0.293	Yes	No
3d	-3.571	94.41	-2.738	-1.076	-2.418	No	0.067	No	-0.35	Yes	No
3e	-3.738	93.138	-2.769	-0.904	-2.176	No	-0.102	No	-0.492	Yes	No
3f	-3.533	94.3	-2.797	-0.922	-2.363	No	-0.136	No	-0.542	Yes	No
3g	-2.987	82.281	-2.735	-1.014	-2.54	No	0.998	Yes	0.221	Yes	No
3h	-3.935	82.358	-2.802	-0.82	-2.455	No	-0.112	Yes	-0.781	Yes	No
3i	-3.77	93.634	-2.996	-.8360	-2.468	No	0.061	No	-0.428	Yes	No
3j	-3.757	93.437	-3.007	-0.833	-2.457	No	-0.009	No	-0.44	Yes	No
3k	-3.748	93.182	-3.01	-0.823	-2.466	No	-0.042	No	-0.463	Yes	No
4a	-3.79	91.409	-3.044	-0.688	-2.391	No	0.211	No	-0.092	Yes	No
4b	-4.184	93.036	-2.912	-0.971	-2.711	No	1.071	No	-0.781	Yes	No
4c	-4.213	90	-3.02	-0.875	-2.282	No	0.172	No	-0.079	Yes	No
4d	-3.963	91.069	-3.101	-0.914	-2.445	No	0.097	No	-0.014	Yes	No
4e	-4.271	89.694	-3.344	-0.824	-2.149	No	0.02	No	-0.104	Yes	No
4f	-3.843	90.829	-3.386	-0.842	-2.335	No	-0.072	No	-0.03	Yes	No
4g	-2.785	73.392	-2.735	-0.675	-2.774	No	0.656	No	0.409	Yes	No
4h	-4.066	82.551	-2.807	-0.828	-2.454	No	-0.044	Yes	-0.579	Yes	No
4i	-3.76	94.307	-3.176	-0.961	-2.512	No	0.105	No	-0.472	Yes	No
4j	-3.754	94.11	-3.213	-0.958	-2.501	No	0.035	No	-0.488	Yes	No
4k	-3.723	93.819	-3.243	-0.946	-2.513	No	0.002	No	-0.53	Yes	No

Abbreviations: WS: water solubility (Log mol/L); ISA: intestinal solubility (%abs); SP: skin permeability (LogKp); BBBP: blood brain barrier permeability (LogBB); CNSP: CNS permeability (LogPS); TC: total clearance (log ml/min/kg); ORAT: Oral Rat Acute Toxicity; HT: hepatotoxicity; SS: skin sensitization.

**Table 4:** Ligand efficacy prediction value.

Ligands	cLog*P*	cLog*S*	LE	LLE	LELP	Mutagenic	Tumorigenic	Irritant
3a	1.65	-2.722	0.4819	7.1314	3.4256	None	None	None
3b	3.3655	-3.319	0.4707	5.1075	7.5279	None	None	None
3c	2.2568	-3.458	0.4562	6.389	4.9469	None	None	None
3d	1.7517	-3.036	0.46204	7.0048	3.7913	None	None	None
3e	2.456	-4.078	0.4724	6.1571	5.1921	None	None	None
3f	1.828	-3.558	0.47949	6.909	3.814	None	None	None
3g	1.6971	-3.726	0.44562	7.0732	3.8084	None	None	None
3h	0.8064	-3.704	0.46204	8.287	1.7453	None	None	None
3i	1.658	-3.262	0.4633	7.1224	3.5787	None	None	None
3j	1.658	-3.262	0.4633	7.1224	3.5787	None	None	None
3k	1.658	-3.262	0.4633	7.1224	3.5787	None	None	None
4a	1.6509	-2.722	0.4819	7.1314	3.4256	None	None	None
4b	3.3655	-3.319	0.44707	5.1075	7.5279	None	None	None
4c	2.2569	-3.458	0.4562	6.3896	4.9469	None	None	None
4d	1.7517	-3.036	0.46204	7.0048	3.7913	None	None	None
4e	2.4532	-4.078	0.47249	6.1571	5.1921	None	None	None
4f	1.8288	-3.558	0.47949	6.909	5.1921	None	None	None
4g	1.6971	-3.726	0.47949	6.909	3.814	None	None	None
4h	0.8064	-3.704	0.44562	7.0732	3.8084	None	None	None
4i	1.658	-3.262	0.46204	8.287	1.7453	None	None	None
4j	1.658	-3.262	0.4633	70122	3.5787	None	None	None
4k	1.658	-3.262	0.4633	70122	3.5787	None	None	None

**Table 5:** Some common amino acid residues involved in interaction with target proteins.

Protein	Common residues involved
CTNNB1	Arg360, ASP361, PRO368, PHE367, MET365
BRCA1	THR1802, CYS1847, HIS1805, ARG1762, ARG1758, ILE1760, LEU1764, ARG1762
BRCA2	PRO924, ASP1122, HIS1061, CYS1060, LEU970, CYS1060, VAL969, ALA1017, GLU1018, ALA874, CYS1060, PHE1016
AKR1C2	LYS270, HIS222, SER217, TRP227, VAL54
ESR	ALA340, VAL534, PRO535, TYR537, SER329, LEU327, GLU330, ALA350, PHE404, LEU391
HER-2	PRO278, ASP8, THR7, THR281, ARG81, LEU414, THR5, SER288
RHEB	ARG7, GLU88, SER179, PRO2, ASN79, MET179
PLAUR	ARG166, LEU181, PRO130, CYS182, ARG230, LEU162, HIS165, SER214
PR	ILE699, ARG766, VAL698, PRO696, MET759, PHE718

**Table 6:** The percentage inhibition of breast cancer cells using MTT assay by the tested compounds at 72 h.

Compounds	% inhibition (*μ*M)				IC_50_
	25	50	100	200	
3a	0.98	15	23.2	48	4.38
3b	5.2	12.6	19.5	39	5.35
3c	6.5	13.6	21.2	32.2	6.23
3d	2.1	9.8	18.5	29.3	6.38
3e	25.6	36.8	54.8	79.4	2.41
3f	28.9	39.6	59.6	77.2	2.41
3g	22.5	33.6	49.9	71.1	2.88
3h	19.9	28.5	46.6	69.3	3.03
3i	11.5	25.3	33.6	58.3	3.69
3j	8.6	19.9	29.6	54.4	3.98
3k	9.7	15.6	27.5	55.9	4.01
4a	0.85	16.5	19.8	32.2	5.85
4b	11.2	21.3	31.5	51.1	4.13
4c	5.5	16.6	28.9	49.2	4.23
4d	6.7	14.9	23.2	35	5.72
4e	21	38.7	66.5	82.2	2.401
4f	28.9	41	69.5	85	2.19
4g	22.6	37.4	59.8	82.3	2.47
4h	24	47.1	61.2	80.01	2.33
4i	19.8	32.5	56.8	75.6	2.699
4j	15.5	28.9	53.6	72.3	2.88
4k	21.3	41.1	66.5	78.9	2.401
Tamoxifen	29.1	55.2	75.4	91.2	1.88

**Table 7:** Chemical structure and corresponding observed and predicted activities obtained from QSARINS.

Compounds	*R*	*R*′	Experimental endpoint	Predicted by model equation	Predicted model equation residual
3a	4-OH	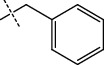	4.38	5.22	2.39
3b	4-OH	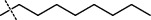	5.35	5.22	-0.12
3c	4-OH	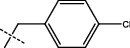	6.23	7.16	0.93
3d	4-OH	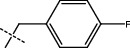	6.38	6.57	0.192
3e	4-OH	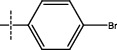	2.41	2.39	-0.015
3f	4-OH	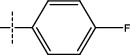	2.41	2.69	-0.035
3g	4-OH	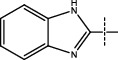	2.88	1.56	-1.31
3h	4-OH	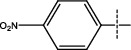	3.03	2.94	-0.08
3i	4-OH	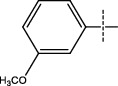	3.69	3.54	-0.14
3j	4-OH	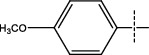	3.98	3.36	-0.6
3k	4-OH	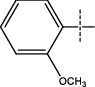	4.01	2.82	-1.18
4a	2-OH	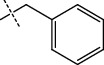	5.85	5.92	0.07
4b	2-OH	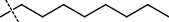	4.13	4.25	0.12
4c	2-OH	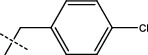	4.23	6.04	1.81
4d	2-OH	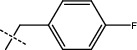	5.72	5.47	-0.24
4e	2-OH	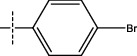	2.40	2.55	0.155
4f	2-OH	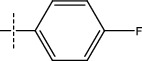	2.19	2.188	-0.0015
4g	2-OH	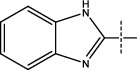	2.47	2.46	-0.003
4h	2-OH	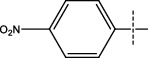	2.33	2.59	0.26
4i	2-OH	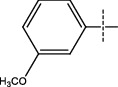	2.69	2.68	-0.013
4j	2-OH	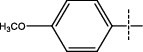	2.88	2.62	-0.25
4k	2-OH	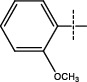	2.40	2.47	0.074

**Table 8:** Pearson correlation matrix.

	MATS3i	VR2_Dzi	ASP-5	GGI10
MATS3i	1.0000			
VR2_Dzi	0.5057	1.0000		
ASP-5	-0.0805	0.2648	1.0000	
GGI10	0.1194	-0.0831	0.0844	1.0000

## Data Availability

All the data has been included in the manuscript.

## Supplementary Materials

Supplementary Materials Supplementary Table 1: the spectral analysis of twenty two derivatives of dihydropyrimidinones.
