# Supplementary material for: Cytotoxic Evaluation, Molecular Docking, and 2D-QSAR Studies of Dihydropyrimidinone Derivatives as Potential Anticancer Agents
Source: J Oncol. 2022 Apr 25;2022:7715689. doi: 10.1155/2022/7715689 (PMC9061032; doi:10.1155/2022/7715689)
Supplement: Supplementary Materials — Supplementary Table 1: the spectral analysis of twenty two derivatives of dihydropyrimidinones. [file 7715689.f1.docx]

| **Supplementary Table 1.** The spectral analysis of twenty two derivatives of dihydropyrimidinones. | | | | | | | |
| --- | --- | --- | --- | --- | --- | --- | --- |
| **Compounds** | **Melting Point** | **FTIR (cm^-1^)** | | | | **^1^H NMR (ppm)** | **^13^C NMR (ppm)** |
|  |  | **OH** | **NH** | **C=C** | **C=O (amide)** |  |  |
| **3a** | 155 | 3590 | 3455 | 1560 | 1690 | 4.10 (s, 2H), 4.33 (s, 2H), 8.10-9.14 (NH, OH) | 50.1 (1C, sp^3^), 115-125 (4C, Ar), 169.2 (1C, C=O) |
| **3b** | 150 | 3600 | 3350 | 1560 | 1680 | 4.37 (s, 2H), 6.65 (d, J=8.19, Ar-2H), 8.08-9.16 (NH,OH) | 14.3-49.6 (8C, sp^3^), 115-125 (4C, Ar), 169.8 (1C, C=O), |
| **3c** | 215 | 3650 | 3490 | 1560 | 1695 | 4.10 (s, 2H), 5.18 (s, 1H), 8.18-9.16 (NH-OH) | 50.1 (IC, sp^3^), 115-125 (4c, Ar), 137.5 (1C, sp^2^), 165.7(1C, C=O) |
| **3d** | 200 | 3595 | 3490 | 1560 | 1695 | 4.19 (s, 2H), 5.19 (s, 1H), 8.8-9.10 (OH, NH) | 50.1 (1C, sp^3^), 52.8 (2C, sp^3^), 168.7 (1C, C=O) |
| **3e** | 220 | 3610 | 3430 | 1560 | 1680 | 4.10 (s, 2H), 9.16 (s, NH, 1H) | 50.1 (1C, sp^3^), 52.8 (1C, sp^3^), 163.9 (1C, C=O) |
| **3f** | 210 | 3600 | 3490 | 1560 | 1690 | 3.9 (s, 2H), 9.06 (s, NH, 1H) | 50.1 (1C, sp^3^), 52.8 (1C, sp^3^), 152.4 (1C, C=O) |
| **3g** | 170 | 3640 | 3090 | 1585 | 1690 | 4.09 (s, 2H), 8.7-9.6 (NH-OH) | 50.1 (1C, sp^3^), 115-125 (8C, Ar), 166.4 (1C, CO-NH) |
| **3h** | 210 | 3630 | 3375 | 1490 | 1700 | 4.10 (s, 2H), 5.10 (s, 1H), 9.16 (s, NH, 1H) | 50.1 (1C, sp^3^), 115-127.4 (8C, Ar), 169.8 (1C, C=O) |
| **3i** | 190 | 3610 | 3490 | 1595 | 1685 | 3.83 (s, 3H, 5.15 (s, 1H), 8-9.04 (NH, OH) | 50.1 (1C, SP^3^), 115-125.3 (8C, Ar), 172.5 (1C, C=O) |
| **3j** | 205 | 3640 | 3455 | 1560 | 1690 | 3.65 (s, 3H), 5.10 (s, 1H), 8.08-9.16 (NH, OH) | 50.1 (1C, sp^3^), 55.5 (1C, OCH3), 170.6 (1C, C=O) |
| **3k** | 215 | 3615 | 3495 | 1500 | 1690 | 3.7 (s, 3H), 5.25 (s, 1H), 9.10 (s, NH, OH) | 50.1 (1C, sp^3^), 55.5 (1C, OCH_3_), 163.4 (1C, CO-NH) |
| **4a** | 195 | 3595 | 3350 | 1585 | 1696 | 4.10 )+(s, 2H), 4.33 (s, 2H), 8.02-9.16 (NH,OH) | 52.8-53.7 (2C, sp^3^), 172.6 (1C, C=O) |
| **4b** | 175 | 3640 | 3100 | 1560 | 1660 | 0.86 (t, J=7.0, 3H), 4.11 (s, 2H), 7.8-9.16 (NH, OH) | 14.3-49.6 (8C, sp^3^), 50.1 (1C, sp3), 167.5 (1C, C=O) |
| **4c** | 210 | 3600 | 3395 | 1535 | 1680 | 4.15 (s, 2H), 5.2 (s, 1H), 8.01-9.06 (NH, OH) | 50.1 (1C, sp^3^), 129.2 (1C, sp^3^), 8.01-9.06 (NH, OH) |
| **4d** | 190 | 3605 | 3495 | 1560 | 1685 | 3.9 (s, 2H), 4.29 (s, 2H), 7.9-9.2 (NH, OH) | 50.4 (1C, sp^3^), 134.1 (1c, Ar), 154.8 (1C, C-OH) |
| **4e** | 175 | 3640 | 3455 | 1600 | 1670 | 4.12 (s, 2H), 5.10 (s, 1H), 7.88-9.05 (NH, OH) | 50.1 (1C, sp^3^), 52.8 (1c, sp^3^), 152.4 (1C, sp^3^) |
| **4f** | 215 | 3610 | 3385 | 1515 | 1690 | 4.20 (s, 2H), 5.20(s, 1H), 7.89-9.16 (NH, OH) | 50.1 (1C, sp^3^), 129.2 (1C, sp^2^), 160.8 (1C, C=O) |
| **4g** | 200 | 3640 | 3455 | 1560 | 1660 | 4.05 (s, 2H), 5.05 (s, 1H), 10.1 (s, NH, 1H) | 52.8 (1C, sp^3^), 137.5 (1C, sp2), 170.8 (1C, C=O) |
| **4h** | 210 | 3590 | 3485 | 1545 | 1690 | 4.10 (s,2H), 5.10 (s,1H), 6.66-8 (m, Ar 8H) | 50.1 (1C), 154.6 (1C, C-OH), 165.8 (1C, C=O) |
| **4i** | 215 | 3640 | 3395 | 1590 | 1695 | 3.9 (s, 3H), 5.18 (s, 1H), 9.02-10.06 (NH, OH) | 50.1 (1C, sp^3^), 55.5 (1C, OCH_3_), 162.8 (1C, C=O) |
| **4j** | 220 | 3640 | 3455 | 1500 | 1680 | 3.68 (s, 3H), 5.15 (s, 1H), 7.9-9.18 (NH,OH) | 50.1 (1C, sp^3^), 55.5 (1C, OCH_3_), 162.8 (1C, C=O) |
| **4k** | 215 | 3625 | 3475 | 1585 | 1680 | 3.75 (s, 3H), 5.16 (s, 1H), 8.01-9.26 (NH, OH ) | 50.1 (1C, sp^3^), 55.5 (1C, OCH_3_), 172.8 (1C, C=O) |
